# VEGF Maintains Maternal Vascular Space Homeostasis in the Mouse Placenta through Modulation of Trophoblast Giant Cell Functions

**DOI:** 10.3390/biom11071062

**Published:** 2021-07-20

**Authors:** Xiujun Fan, Shanmugam Muruganandan, Philemon D Shallie, Sabita Dhal, Matthew Petitt, Nihar R Nayak

**Affiliations:** 1 Laboratory of Reproductive Health, Shenzhen Institute of Advanced Technology, Chinese Academy of Sciences, Shenzhen 518055, China; 2 Department of Obstetrics and Gynecology, Wayne State University School of Medicine, Detroit, MI 48201, USA; m.shanmugam@northeastern.edu (S.M.); nnayak@umkc.edu (N.R.N.); 3 Department of Biology, Northeastern University, Boston, MA 02115, USA; 4 Department of Obstetrics and Gynecology, UMKC School of Medicine, Kansas City, MO 64108, USA; pdsxfn@umkc.edu (P.D.S.); sdcm7@umkc.edu (S.D.); petitt@mac.com (M.P.)

**Keywords:** placenta, VEGF, sFlt1, TGC

## Abstract

Vascular endothelial growth factor (VEGF) is an angiogenic growth factor that acts primarily on endothelial cells, but numerous studies suggest that VEGF also acts on non-endothelial cells, including trophoblast cells. Inhibition of VEGF signaling by excess production of the endogenous soluble VEGF receptor sFlt1 in trophoblast cells has been implicated in several pregnancy complications. Our previous studies and other reports have shown that VEGF directly regulates placental vascular development and functions and that excess VEGF production adversely affects placental vascular development. Trophoblast giant cells (TGCs) line the maternal side of the placental vasculature in mice and function like endothelial cells. In this study, we specifically examined the effect of excess VEGF signaling on TGC development associated with defective placental vascular development using two mouse models an endometrial VEGF overexpression model and a placenta-specific sFlt1 knockdown model. Placentas of endometrial VEGF-overexpressing dams at embryonic days (E) 11.5 and 14.5 showed dramatic enlargement of the venous maternal spaces in junctional zones. The size and number of the parietal TGCs that line these venous spaces in the placenta were also significantly increased. Although junctional zone venous blood spaces from control and VEGF-overexpressing dams were not markedly different in size at E17.5, the number and size of P-TGCs were both significantly increased in the placentas from VEGF-overexpressing dams. In sFlt1 knockdown placentas, however, there was a significant increase in the size of the sinusoidal TGC-lined, alkaline phosphatase-positive maternal blood spaces in the labyrinth. These results suggest that VEGF signaling plays an important role in maintaining the homeostasis of the maternal vascular space in the mouse placenta through modulation of TGC development and differentiation, similar to the effect of VEGF on endothelial cells in other vascular beds.

## 1. Introduction

Vascular endothelial growth factor (VEGF) is an angiogenic factor widely studied for its critical and highly dose-sensitive role in embryonic vascular development. Although VEGF primarily targets endothelial cells [1,2,3], it can also target various non-endothelial cells, including placental trophoblasts, which have endothelial cell characteristics [4,5]. Unlike the vascular spaces of other tissues, the maternal blood spaces in the placenta are not lined by endothelial cells, but rather by a variety of specialized trophoblast cells that, in mice, are called trophoblast giant cells (TGCs) [5,6,7]. Previous studies have identified multiple subtypes of TGC based on morphological and molecular characteristics [5,6,7,8]. These include spiral artery-associated TGCs (SpA-TGCs), maternal blood canal-associated TGCs (C-TGCs), sinusoidal TGCs (S-TGCs), TGCs lining the small channels that collect deoxygenated blood from the labyrinth, called channel TGCs (Ch-TGCs), and the TGCs surrounding the lacunae that channel maternal blood into the uterine veins, called parietal TGCs (P-TGCs) [5,6,7,8]. The SpA-TGCs invade the spiral arteries to regulate maternal blood flow into the placenta. Spiral arteries converge to form straight canals lined by C-TGCs at the border of the decidua and the junctional zone. The canals, in turn, lead into sinusoidal spaces in the labyrinth, which harbors S-TGCs and ultimately drains into the venous lacunae, lined by P-TGCs, through channels lined by Ch-TGCs [5,6,7,8,9]. Although TGCs mimic most of the cellular and molecular functions of endothelial cells in other vascular beds, the role of VEGF in the development and functions of TGCs in the placental maternal vascular space is not fully understood.

Just as appropriate levels of VEGF signaling in endothelial cells are critical for normal vascular development, appropriate levels of VEGF signaling in placental trophoblasts have been shown to be critical for normal placental development in mice [1,2,5,8,9]. The majority of VEGF signaling in vascular endothelial cells occurs through VEGF receptor 2 (VEGFR2), but vascular endothelial cells also express the membrane-associated and soluble forms of VEGF receptor 1 (VEGFR1), also termed fms-like tyrosine kinase 1 (Flt1) [5,8,9]. Soluble Flt1 (sFlt1) binds to VEGF-A and acts as a decoy receptor to control local levels of VEGF signaling [10,11,12]. Placental trophoblasts also express sFlt1, which plays an essential role in modulating the VEGF signal from the maternal endometrium [8,9,12,13]. Excess sFlt1 production during pregnancy in humans is associated with serious pregnancy complications, including preeclampsia, a major cause of maternal and fetal morbidity and death [8,9,14,15,16]. Understanding the role of VEGF signaling in pregnancy therefore has implications for understanding not only placental development but also the pathogenesis of pregnancy complications.

Our previous studies and other reports in mice have shown that VEGF directly regulates placental vascular development and functions and that excess VEGF signaling in the placenta interferes with normal placental vascular development [9,13,14,15,16]. Targeted overexpression of VEGF in the maternal endometrium and targeted knockdown of sFlt1 in placental trophoblasts have both been shown to adversely affect the development of maternal vascular spaces, causing enlargement of maternal blood spaces, suggesting potential effects on TGC development and/or function [8,9]. In this study, we examined the specific effects of increased VEGF signaling on TGC development and differentiation using our previously developed endometrial VEGF overexpression and placenta-specific sFlt1 knockdown models [9]. Our findings indicate that different TGC subpopulations respond differently to increased VEGF signaling and provide evidence for differences in the dynamics of VEGF signaling in different regions of the placenta.

## 2. Methods

### 2.1. Animals

Procedures involving mice were approved (Protocol number 12340) by the Administrative Panel on Laboratory Animal Care of Stanford University, Palo Alto, CA, USA. Mice were kept on a 12:12 h light–dark cycle and allowed free access to feed and water. To induce pregnancy or pseudopregnancy, 8–12 weeks CD-1 female mice (Charles River) were mated with fertile or vasectomized males (10–16 weeks of age). Mating was begun in the evening, and females were examined for the presence of viscous vaginal plugs the next morning. Noon on the day a vaginal plug was detected was designated gestation day E0.5. Pregnant females were euthanized, and placentas were harvested at E11.5, E14.5, or E17.5.

#### 2.1.1. Endometrium-Specific VEGF Overexpression in Mice during Pregnancy

To generate endometrium-specific VEGF-overexpressing mice, we employed the lentiviruses LV-VEGF/GFP and LV-Fluc/GFP for the delivery of VEGF and Fluc expression cassettes [9], as described in our previous studies [9,17]. Briefly, uterine horns were denuded, removing luminal epithelium, prior to the viral transduction procedure. After denudation, 100 µL of PBS containing LV-Fluc/GFP (1 × 10^10^ particles) or LV-VEGF/GFP (1 × 10^10^ particles) were infused into the lumen of each uterine horn from the oviductal end using a 31G needle. To examine transgene expression, uteri from LV-Fluc/GFP-injected mice were excised, and the cellular localization of GFP was determined by IHC. Data confirming transgene expression are presented in our previous study [9,17]. For both LV-VEGF/GFP and LV-Fluc/GFP, mice were allowed to mate five days after virus infusion.

#### 2.1.2. Placenta-Targeted Expression of sFlt1 shRNA

Knockdown of placental sFlt1 expression was achieved by lentivirus-mediated RNA interference as described previously [9]. As presented in our earlier work, we used LV-copGFP as a control and LV-sFLT1shRNA-copGFP for expressing sFLT1 shRNA in mouse blastocysts. Detailed procedures for determining the optimal virus titers, transduction protocols, selection of transduced blastocysts, and transfer of blastocysts into pseudopregnant mice are described in our earlier studies [9,17,18]. Briefly, individual blastocysts from pregnant mice were collected and transduced with LV-sFlt1shRNA-copGFP or LV-copGFP after removal of zona pellucidae. Blastocysts that expressed GFP after transduction were used for subsequent transfer into pseudopregnant CD1 mice. Placenta-specific expression of sFLT1 shRNA was verified by measuring reduction in placental sFLT1 mRNA by in situ hybridization (ISH) and quantitative real-time PCR (qPCR), whereas reduction in levels of sFLT1 protein were verified by Western blotting and presented in our earlier study [9].

### 2.2. Histology

Placental tissues were collected and processed for histological analysis according to procedures described earlier [9]. Briefly, placental samples were collected on E11.5, E14.5, or E17.5, fixed in 4% paraformaldehyde overnight at 4 °C, embedded in paraffin, sectioned, and stained with H&E.

### 2.3. Alkaline Phosphatase Histochemical Staining

Alkaline phosphatase (AP) has been shown to be specifically expressed by the trophoblast cells lining the maternal blood spaces and serves as a marker for these cells [19]. We therefore used AP expression to identify TGCs, as described in previous studies [9,19]. Paraffin sections of 5 μm thickness were dewaxed, rehydrated, and washed in NT solution (0.15 M NaCl and 0.1 M Tris, pH 7.5) for 20 min at room temperature followed by a second wash with freshly prepared NTMT solution (0.1 M NaCl, 0.1 M Tris, pH 9.5, 0.05 M MgCl_2_, and 0.1% Tween-20) for 10 min at room temperature. AP activity was visualized after incubation with a standard chromogenic AP substrate (BCIP/NBT; Promega), counterstaining with Nuclear Fast Red, dehydrating by passing through a graded ethanol series, and mounting after clearing in xylene [9,19].

### 2.4. Image Processing and Morphometric Analysis

TGC number and diameter were quantified using ImageJ (IJ1.46r, NIH) software. MBS diameters were measured from AP histochemically stained sections using AxioVision 3.0 software. At least 3 different sections for each mouse were used in all quantifications. Mean MBS diameters were calculated from multiple measurements of each MBS.

### 2.5. Statistical Analysis

Data were analyzed using GraphPad Prism 5.0 software and expressed as mean ± standard deviation. Comparisons between values were performed using a Student’s *t*-test (two-tailed). For all statistical analyses, *p* < 0.05 was considered statistically significant.

## 3. Results

### 3.1. Endometrial VEGF Overexpression Leads to Dramatic Alteration of Parietal and Sinusoidal Trophoblast Giant Cell Differentiation and Abnormal Enlargement of Maternal Blood Spaces in the Placenta

We previously reported the development of mouse models for endometrial overexpression of VEGF and placenta-specific knockdown of sFlt1 [9]. Both of these manipulations were found to cause dramatic changes in placental blood spaces, suggesting that VEGF signaling has direct effects on these blood spaces. We therefore sought to further characterize the specific effects of these treatments on placental blood spaces and TGCs that line the maternal aspect of these spaces.

**Figure 1:**
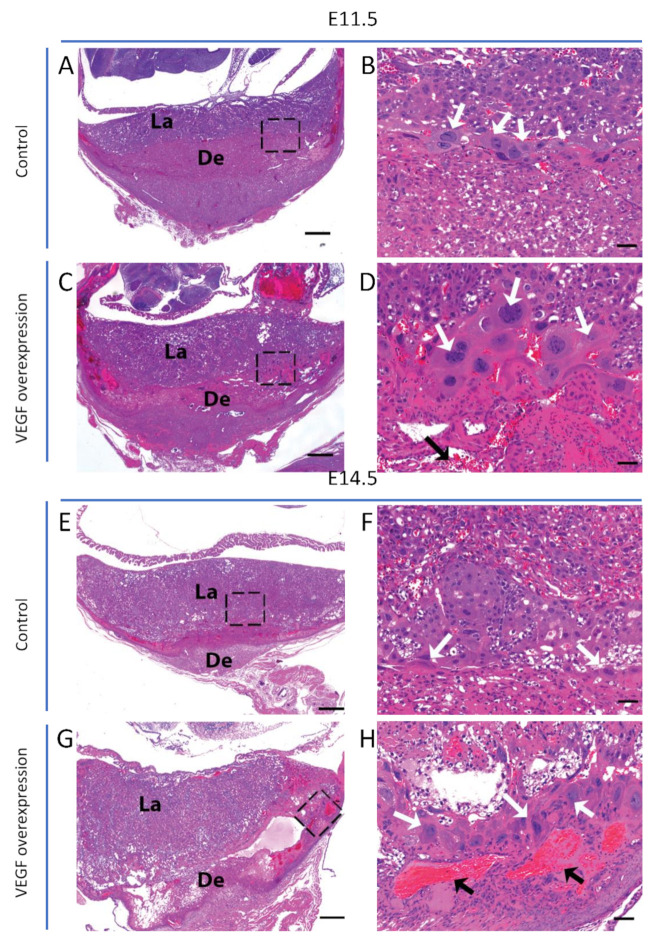
**Alteration of placental vascular spaces and associated TGC differentiation in endometrial VEGF-overexpressing dams.** Placental sections stained with H&E at E11.5 (**A**–**D**) and E14.5 (**E**–**H**). Histological sections of placentas from endometrial VEGF-overexpressing dams (**C**,**G**) showing severely dilated maternal blood spaces (indicated by black arrows within the insets, **D**,**H**) and increased number and size of TGCs (indicated by white arrows within the insets, **D**,**H**) as compared to the control (**A**,**B**,**E**,**F**). De, decidua; La, labyrinth; TGC, trophoblast giant cells. Scale bar in A, **C**,**E**,**G** = 500 µm. Scale bar in **B**,**D**,**F**,**H** = 50 µm.

**Figure 2:**
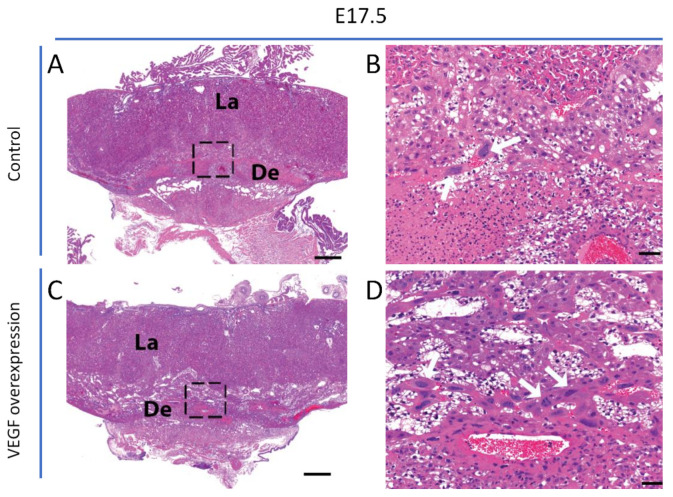
**Pregnancies at term exhibit less impact on TGCs.** Full-thickness H&E-stained placentas at E17.5 (**A**–**D**). Control dams exhibiting normal placental development at E17.5 (**A**,**B**). Placentas surviving to term in endometrial VEGF-overexpressing dams at E17.5 showing changes in TGCs (indicated by white arrows within the insets, **C**,**D**) but no discernible effect on maternal blood spaces in the placenta (**C**,**D**) compared to the controls (**A**,**B**). De, decidua; La, labyrinth; TGC, trophoblast giant cells. Scale bar in **A** and **C** = 500 µm. Scale bar in **B** and **D** = 50 µm.

**Figure 3:**
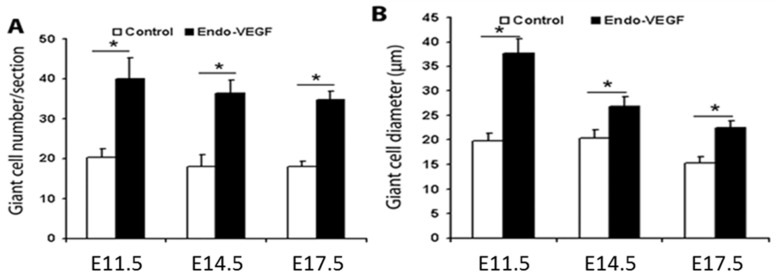
**Increased number and size of TGCs in endometrial VEGF-overexpressing dams.** Quantitative analysis of TGC number (**A**) and size (diameter, **B**) at E11.5, E14.5, and E17.5. Mean number of TGCs measured from control placentas (white bars) and placentas derived from endometrial VEGF-overexpressing dams (black bars) at E11.5, E14.5, and E17.5. Error bars indicate standard deviation. Asterisks (*) denote statistically significant differences between groups (*p* < 0.05). n = 10 placentas taken from dams treated with lentiviruses expressing GFP (control) and those expressing VEGF (Endo-VEGF).

In mice overexpressing VEGF in the endometrium, junctional zone venous maternal blood spaces were enlarged at E11.5 and E14.5 relative to those in control placentas (Figure 1). Analysis of TGCs revealed that the cellularity and size of TGCs increased in endometrial VEGF-overexpressing dams (Figure 1). The number and size of TGCs at E17.5 (Figure 2) were also increased; however, these changes were not as dramatic as those observed at E11.5 and E14.5, consistent with the finding that surviving placentas at E17.5 showed less overt phenotypes from endometrial VEGF overexpression. ImageJ analysis and quantification of the abundance (number) and sizes (diameter) of parietal TGCs that line the maternal blood spaces in the junctional zone reveal that endometrial overexpression of VEGF significantly increases the number of TGCs in the junctional zone at E11.5 and E14.5 that also persists in the placentas surviving the VEGF overexpression at E17.5 gestational age (Figure 3). However, the maternal blood spaces in the labyrinth zone of placentas from VEGF-overexpressing mice appeared normal, without any obvious abnormalities in the sinusoidal trophoblast giant cells that line these vascular spaces (Figure 1). These findings suggest that endometrial VEGF overexpression directly influences the differentiation of parietal TGCs in the junctional zone. As shown in our previous report [9], endometrial VEGF overexpression in these mice leads to a reduction in the number of fetuses and an increase in the number of resorption sites, suggesting that conceptuses and placentas with more severe developmental defects do not survive pregnancy and that those observed at E17.5 represent those less severely affected by endometrial VEGF overexpression. Consistent with this less severe effect in pregnancies continuing to term, the venous blood spaces in the junctional zone at E17.5 appeared largely normal (Figure 2).

### 3.2. Placenta-Specific Knockdown of sFlt1 Preferentially Alters Sinusoidal Trophoblast Giant Cell Morphology and Number

**Figure 4:**
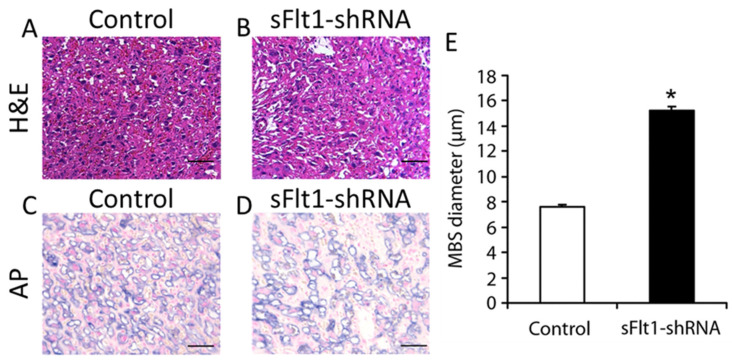
**Expanded maternal sinusoidal blood spaces in the labyrinths of sFlt1-knockdown placentas.** H&E-stained histological sections of placentas derived from placenta-specific sFlt1 knockdown dams showing severely dilated maternal blood spaces as compared to those in control placentas (**A**–**B** H&E). Alkaline phosphatase staining indicates the dilatation of maternal blood spaces in the placenta (**C**,**D**, AP). **E**, Maternal blood space (MBS) diameters. Mean MBS diameters in control placentas (white bar) and placentas derived from placenta-specific sFlt1 knockdown dams (black bar) at E17.5. Error bars indicate standard deviation. Asterisks (*) denote statistically significant differences between groups (*p* < 0.05). n = 15 each for placentas taken from dams expressing copGFP alone (control) and those expressing sFlt1 shRNA plus copGFP (sFlt1-shRNA). Scale bar = 50 µm.

The above observations on the effects of endometrial VEGF overexpression on the maternal vascular spaces indicate that endometrial VEGF has different effects on the junctional zone and labyrinth. Differences in local VEGF concentrations between these two regions likely contribute to the differential effects. One reason for differences in local VEGF levels could be related to the location of the junctional zone, which is more proximal to the source of endometrial VEGF, enabling higher VEGF concentrations and greater changes compared to those in the labyrinth. Additionally, the labyrinth zone is believed to be protected from the adverse effects of excess VEGF by stimulation of sFlt1 production in the junctional zone cells, neutralizing the VEGF from maternal sources [9]. Therefore, to further explore the mechanisms by which increased VEGF signaling in the placenta may influence placental vascular spaces, we knocked down sFlt1 expression specifically in the placenta using our previously described placenta-specific shRNA mouse model and examined the effects in placentas at E17.5 (Figure 4). Histological analysis of the labyrinth revealed a dramatic increase in the size of blood spaces (Figure 4A,B, H&E). To determine whether these sinusoidal blood spaces were of maternal or fetal origin, we performed alkaline phosphatase staining to identify maternal sinusoidal blood spaces (Figure 4C,D, AP). Image analysis and quantification of maternal blood space (MBS) diameters revealed that the sinusoidal trophoblast giant cell (sTGC)-lined maternal blood spaces in the labyrinths of placentas derived from sFlt1 knockdown mice were significantly larger than those in control placentas (Figure 4E).

## 4. Discussion

We previously showed that local increases in VEGF signaling in the mouse placenta during pregnancy—either through endometrium-specific overexpression of VEGF or placenta-specific knockdown of sFlt1—adversely impacts placental vascular development and homeostasis [9]. We hypothesized that these changes resulted from the effects of excess VEGF signaling on differentiation of TGCs in different zones during placental development, leading to developmental and phenotypic changes in these cells. Our results indicate that VEGF affects the development of maternal blood spaces by regulating TGC numbers and differentiation. Several previous studies have shown that trophoblast stem cells can differentiate into a variety of TGC subtypes [9,20,21,22], and TGCs can form vascular-like cavities in suspension cultures [22]. VEGF is also known to stimulate lumen formation by endothelial cells during vascular development [23]. Future studies will assess specific VEGF signaling pathways involved in differentiation of specific subtypes of TGCs.

We found that excess VEGF production in the maternal decidua preferentially affected parietal TGCs lining venous maternal blood spaces in the junctional zone and was associated with expansion of these spaces, whereas spiral arteries and canals were not detectably changed [9]. This suggests differences in responsiveness to VEGF between different TGC subtypes, perhaps due to differences in the expression of VEGF receptors or differences in downstream signaling capabilities. Previous studies have also reported different types of progenitor cells in the junctional zone that give rise to different TGC subtypes [6,7,24], suggesting that different trophoblast progenitors may respond differently to changes in VEGF levels.

Our results also indicate that endometrial VEGF overproduction and placental sFlt1 knockdown preferentially affect different TGC subpopulations, with VEGF overproduction primarily affecting the junctional zone venous maternal blood spaces and parietal TGCs and placental sFlt1 knockdown preferentially affecting sinusoidal spaces and S-TGCs in the labyrinth. This difference may reflect the difference in anatomical origins of altered expression. VEGF signaling along the endometrial–placental axis might be expected to be greatest on the endometrial side (i.e., junctional zone), as endometrial VEGF is increasingly neutralized by local placental sFlt1 production toward the placental pole. As the placenta increases its production of sFlt1 in response to increased endometrial VEGF, abnormal levels of VEGF signaling may occur in the more endometrium–proximal junctional zone but have an increasingly smaller effect on deeper parts of the placenta and the fetus as VEGF is sequestered, consistent with the hypothesis that increases in sFlt1 production in response to VEGF serve to protect the placenta and the fetus from toxic levels of VEGF signaling [9]. Conversely, placental knockdown of sFlt1 would be expected to enable greater penetration of endometrial VEGF into the deeper placental zones and the fetus itself, consistent with our observation that sFlt1 knockdown leads to increased fetal loss and developmental effects on the placenta preferentially in the labyrinth. In normal pregnancy, sFlt1 may play a lesser role in limiting VEGF activity in the junctional zone, but a critical role in limiting VEGF activity in the labyrinth and fetus [9].

VEGF has two major receptors: VEGF receptor 1 (VEGFR1, or Flt1) and VEGF receptor 2 (VEGFR2). Although the majority of VEGF-A signaling in endothelial cells occurs through VEGFR2, VEGFR2 has not been widely detected in trophoblasts. Nonetheless, mouse trophoblasts are strongly affected by VEGF overexpression, altering normal development and upregulating sFlt1 [9]. In contrast to VEGFR2, VEGFR1 is widely expressed in trophoblasts. There is evidence that VEGFR1 can act as a signaling receptor in endothelial cells [25,26], but the majority of *Vegfr1* transcripts in trophoblasts encode the soluble form of Flt1 (sFlt1). Although *Vegfr1* is not required for the establishment of the maternal-fetal interface at early stages of pregnancy [27], it remains unclear which receptors mediate the effects of increased VEGF signaling on placental trophoblasts.

## 5. Conclusions

In summary, this study has provided new insights into the role of VEGF signaling in maintenance of the homeostasis of the placental maternal vascular space through modulation of TGC development and differentiation. Our results indicate not only that VEGF is a regulator of placental TGC differentiation and proliferation, but also that it differentially affects different subpopulations of TGCs. Our results also support the notion that sFlt1 serves to protect the fetus and fetal-proximal regions of the placenta from abnormal levels of VEGF signaling that can damage pregnancy [9]. Further studies are needed to understand why TGC subpopulations that line the spiral arteries or canals appear to be more refractory to the overexpression of VEGF, perhaps due to a reduced level of active VEGF receptors or greater local production of sFlt1.

## Abbreviation

BCIP	5-Bromo-4-chloro-3-indolyl phosphate
Ch-TGCs	Channels TGCs
C-TGCs	Canal TGCs
E	Embryonic days
Flt1	fms-like tyrosine kinase 1
Fluc	Luciferase
GFP	Green Fluorescent Protein
H&E	Hematoxylin and Eosin
LV	Lentiviruses
NBT	Nitro blue tetrazolium
NT	Neutralize Tagment buffer
NTMT	Alkaline phosphatase buffer
P-TGCs	Parietal TGCs
sFlt1	Soluble Flt1
shRNA	A short hairpin RNA or small hairpin RNA
SpA-TGCs	Spiral artery-associated TGCs
TGCs	Trophoblast giant cells
VEGF	Vascular endothelial growth factor
VEGFR1	Vascular endothelial growth factor 1 Receptor
VEGFR2	Vascular endothelial growth factor 2 Receptor
